# Postoperative Pain After Using Reciprocating Motion with Reciproc Files Versus Adaptive Motion with Twisted File Adaptive in Instrumentation of Necrotic Mandibular Molars: A Randomized Clinical Trial

**DOI:** 10.14744/eej.2021.53215

**Published:** 2022-03-14

**Authors:** Ahmed Yaser ABU BAKR, Hebatallah Mohamed MAGED ELFAR, Ghada El Hilaly MOHAMED EID

**Affiliations:** From the Department of Endodontics, (A.Y.A.B., 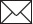 ahmed.abubakr@dentistry.cu.edu.eg, H.M.M.E., G.E.H.M.E.), Cairo University, Faculty of Dentistry, Giza, Egypt

**Keywords:** Nickel-titanium instruments, postoperative pain, reciproc, TF adaptive

## Abstract

**Objective:**

The objective of the present study was to assess the effect of using Reciproc (RC), (VDW GmbH, Munich, Germany) files with reciprocating motion versus Twisted File Adaptive (TFA, Kerr, Orange, California, USA) system with adaptive motion on post-instrumentation and post-obturation pain of necrotic mandibular molars.

**Methods:**

Fifty-eight patients with mandibular molar assessed at 3 intervals; 6, 12 and, 24 hrs. Mann Whitney U and Friedman test was used for data analysis, and the significance level was set to (P≤0.05).

**Results:**

There was no statistically significant difference in the mean values of post-instrumentation pain at each time interval for the RC and TFA groups (P>0.05). Pain decreased in each group with a statistically significant difference from preoperative condition to all six post-instrumentation time intervals (P<0.001). In each group, post-instrumentation mean pain values at 6, 12, and 24 hrs were higher than post-obturation pain values at 6, 12, and 24 hrs with a statistically significant difference (P<0.001).

**Conclusion:**

TFA and RC had a similar impact on post-instrumentation and post-obturation pain. The post-instrumentation pain was higher than post-obturation pain in both groups.

**HIGHLIGHTS**

• This study evaluated pain after instrumentation and obturation using RC and TFA files to decrease this complication.
• There was no significant difference in post-instrumentation and post-obturation pain between RC and TFA groups.
• Post-instrumentation pain was higher than post-obturation pain.

## INTRODUCTION

Postoperative pain is a common complication after root canal treatment and in-between visits, where necrotic canals present more challenges than vital diseased pulps (1). Dentinal debris, microorganisms, and irrigants may be introduced into the periapical tissues during chemomechanical preparation, causing apical inflammation and postoperative pain (1). Instrumentation techniques with different kinematics are associated with variable apical extrusion of debris (2) and may contribute to post-instrumentation pain. Most nickel-titanium (NiTi) engine-driven systems extrude less debris than manual stainless-steel K-files manipulated by hand because of their rotary action, which can remove coronal debris (3); thus, they may have the potential to reduce the risk of postoperative discomfort.

Single file, reciprocating NiTi systems such as Reciproc (RC) and WaveOne (Dentsply Sirona, Ballaigues, Switzerland), made of M-Wire, were introduced to the market with advantages of increasing flexibility, resistance to flexural cyclic fatigue, and decreasing operating time compared to rotary instruments (4, 5). RC is a single-file system with an S-shaped cross-section and spiral flutes with high cutting efficiency and a gradually decreasing taper after the apical 3 mm. It features a specialised motor that performs alternating counterclockwise (CCW) 150° (cutting angle) to cut dentine and clockwise (CW) 30° (releasing angle) to release the file from the canal wall. A systematic review by Martins et al. (6) revealed reciprocating instruments lead to lower postoperative pain than rotary instruments. Neelakantan et al. (7) reported that RC caused less postoperative pain after a single visit than One Shape single rotary NiTi file with a statistically significant difference. Oliveira et al. (8) found no significant difference in mean Visual Analogue Scale (VAS) pain scores between RC and ProTaper Next after root canal instrumentation in two-visit root canal treatment. Accordingly, RC did not increase postoperative pain compared to other rotary instruments (6-8).

Twisted File Adaptive (TFA) is a multi-file system made of R-phase NiTi, with a triangular cross-section utilising a combination of rotation and reciprocating motions. When the file is very lightly stressed, the movement can be described as continuous interrupted rotation to allow a better cutting efficiency and debris removal than continuous rotation (9, 10). When a load is applied, the endodontic motor reciprocates from 600° to 370° clockwise, and 0° to 50° counterclockwise, dependent on the torsional resistance sustained on the file (11). The adaptive motion combines both rotation and reciprocation advantages, decreasing the debris extrusion (10) and reducing postoperative pain (10). Manufacturers claimed that this adaptive technology, combined with the flexibility of the TF, could allow the file to adjust to intracanal torsional forces depending on the amount of pressure placed on the file. In addition, it is reported that compared with a single reciprocating file system (WaveOne in single-visit treatment), TFA files caused less postoperative pain (10).

Only a single study (8) assessed pain after instrumentation using RC, and no study reported post-instrumentation pain after using TFA. To date, no randomised clinical trials have been conducted to evaluate reciprocating motion versus adaptive motion on both post-instrumentation and post-obturation pain in two-visit treatment. Thus, the purpose of the current study was to assess the effect of using RC files versus TFA on pain after instrumentation of necrotic mandibular molars. The null hypothesis was that there was no difference between the two instrumentation techniques. 

## MATERIALS AND METHODS

This study was approved by the Department of Endodontics, evidence-based committee, postgraduate committee, and Ethics Committee in December 2017. In addition, the protocol was registered on Clinical Trials.gov (Identifier: NCT03338322).

**Sample size:** Sample size calculation was performed using PS: Power and Sample Size Calculation Software Version 3.1.2 (Vanderbilt University, Nashville, Tennessee, USA). The sample size was based on a previous study by Zand et al. (2016) (12) with α=0.05 and a power of 80%. A total of 58 patients were enrolled in the present study.

### Patient selection

Fifty-eight patients (43 females and 15 males with an age range of 18-60 years) with mandibular molar teeth diagnosed with necrotic pulps with symptomatic or asymptomatic apical periodontitis were included in the study. Patients were attending the endodontic clinic, Faculty of Dentistry. Patients under analgesic medication 12 hrs before the treatment, patients with systemic disease, or those with localised vestibular swellings or cellulitis were not included in the study.

Included patients had no pain with thermal changes. Necrotic pulps were diagnosed as teeth with no response to cold testing using ice sticks and cold-water bath after isolation with a dental dam. The presence of apical periodontitis was determined by the patient having pain on biting. Clinically, if the tooth was sensitive to palpation and percussion, it was diagnosed with symptomatic apical periodontitis. In contrast, if it had a normal response, it was diagnosed as having asymptomatic apical periodontitis. The odontogenic cause was confirmed by the presence of deep restorations, caries, or pulp exposure. Radiographic confirmation of apical periodontitis was the presence of periapical radiolucency or slight widening of periodontal ligament space.

**Pain assessment:** A numerical rating scale (NRS) (13) was used to score clinical pain intensity in three procedural steps: diagnosis, post-instrumentation, and post-obturation phases. Each patient rated their pain level from 0 to 10. Afterwards, pain intensity was defined as 0 reading represented (no pain), 1 to 3 reading represented (mild pain), 4 to 6 reading represented (moderate pain), and 7 to 10 reading represented (severe pain).

**Radiographic examination:** Periapical radiographs were obtained with parallel technique using conventional films (D speed x-ray film, Carestream dental, New York, USA), film holder (Dentsply Rinn, Elgin, IL), and an x-ray tube (Belmont BelRay II, Takara company, Canada) at 60 kV and 7 mA and intervals ranging from 0.25 to 0.32 sec depending on bone density.

**Random selection of instrumentation system:** This study had a two parallel-arm design with random allocation into groups using random.org software. Allocation was concealed up to the instrumentation step as the investigator would phone the supervisor to enrol the patient according to the sequence generation. The supervisor kept a random sequence table, and the first investigator was responsible for the enrolment and treatment of eligible patients. The groups were allocated as follows: In the reciprocating group (n=29), instrumentation was done by reciprocation motion using RC. In the adaptive motion group, instrumentation was performed using TFA (n=29). 

**Treatment protocol:** After the procedures of the study were explained to the patients, they signed informed consent.

**At the first visit:** An inferior alveolar nerve block was administered using 1.8 mL of 2% lidocaine (Lidocaine, Safco Dental Supply Co., Buffalo, NY, United States) containing 1:100,000 epinephrine. The access cavity was prepared using a round bur size 3 and an Endo Z bur (Mani Inc., Utsunomiya, Japan). After the tooth was isolated with a dental dam, initial negotiation of the canal was done using conventional stainless steel hand instrument K-files size 10 (Mani Inc.) followed by K-file size 15. Finally, the working length determination was performed with an apex locator (Root ZX II, J Morita Corp, Kyoto, Japan) and was confirmed radiographically.

**Instrumentation in the RC group:** RC files were used under reciprocation according to manufacturer recommendations (X-smart plus, Dentsply-Sirona, Ballaigues, Switzerland). R25 (25, 8% variable regressive taper) was used in narrow and/or curved canals where SS K-file size 15 would fit passively, R40 (40, 6% variable regressive) was used in large and curved canals where SS K-file size 20 would fit passively, and R50 (50, 5% variable regressive) was used where SS K-file size 30 would fit passively inside the canal. Files were used by three in and out motions with strokes not exceeding 3 mm in length advancing from cervical until obtaining the entire working length (14). Irrigation with 2 mL 5.25% sodium hypochlorite (NaOCl) with a rate of 0.1 mL/3 sec was performed between each stroke using a side-vented needle 30 gauge located at a maximum of 1 mm from working length. Instruments were frequently cleaned by moistened gauze to eliminate debris. Ethylenediaminetetraacetic acid, 17% (EDTA) gel, (MD-ChelCream^TM^, Meta Biomed CO.LTD, Chungbuk, Republic of Korea) was used as a lubricant on each file before insertion in canals.

**Instrumentation in the TFA group:** TFA files were used with adaptive motion. The TFA setting was used on the Elements motor (Kerr, Orange, California, USA). According to manufacturer recommendations, files were used with a single controlled motion with slow advancement of the file inside the canal. Next, the file was withdrawn, and its flutes were wiped, followed by 5.25% NaOCl irrigation. This step was repeated until reaching its full working length. Narrow and curved canals were instrumented with SM files (20, 4%; 25, 6% and 35, 4%). Medium or large canals were instrumented with ML files (25, 8%; 35, 6% and 50, 4%).

**Preparation time:** The preparation time for both groups was recorded for each case using a stopwatch. The duration of instrumentation included the total active instrumentation of all canals, time taken for file replacement, irrigation between files, and cleaning the flutes of the rotary files.

After instrumentation, canals were dried with paper points, and the access was closed with a cotton pellet inside the pulp chamber and sealed by temporary filling (MD Temp, Meta Biomed CO.LTD, Chungbuk, Republic of Korea) until the next visit with confirmation of coronal seal, absence of occlusal interference and gingival impingement.

**At the second visit:** A dental dam was applied and the temporary filling was removed. Irrigation was performed with 20mL 5.25% NaOCl using a side-vented 30 gauge needle.

**Obturation:** The canals were dried with paper points, and master cones were checked clinically and radiographically. Lateral compaction obturation was performed using matching gutta-percha sizes of 25, 8% or 40, 6% or 50, 5% in cases prepared by RC and size 35, 4% or 50, 4% in cases prepared by TFA. A size 30 spreader and size 25 auxiliary cones and resin sealer (ADSEAL, Meta Biomed CO.LTD, Chungbuk, Republic of Korea) were used.

After obturation, a cotton pellet was placed in the pulp chamber, and the access cavity was closed with a temporary filling to avoid coronal leakage. The patient was referred to the restorative department. 

**Assessment of outcomes:** The primary outcome of post-instrumentation pain was measured by (NRS) at 6, 12, 24, 48, 72 hrs, and 1 week after instrumentation. The secondary outcome of post-obturation pain was measured by (NRS) at 3 intervals; 6, 12, and 24 hrs after obturation. Furthermore, the duration of instrumentation and the number of analgesic tablets taken by the patient during 1^st^ week after instrumentation and during the first 24 hrs after obturation were recorded. 

The patients were asked to mark the score representing their pain level on NRS. Phone calls were made to remind patients to mark their scores. Then, the operator collected the data from patients as they brought them back.

### Statistical analysis

Data were analysed using IBM SPSS advanced statistics (Statistical Package for Social Sciences), version 21 (SPSS Inc., Chicago, IL, USA). Data were explored for normality using the Kolmogorov-Smirnov test and Shapiro-Wilk test. Comparisons between two groups were made using the student's t-test and the Mann-Whitney test. Pain over time was assessed with Friedman's test. A P-value less than or equal to 0.05 was considered statistically significant.

## RESULTS

**Figure 1.**
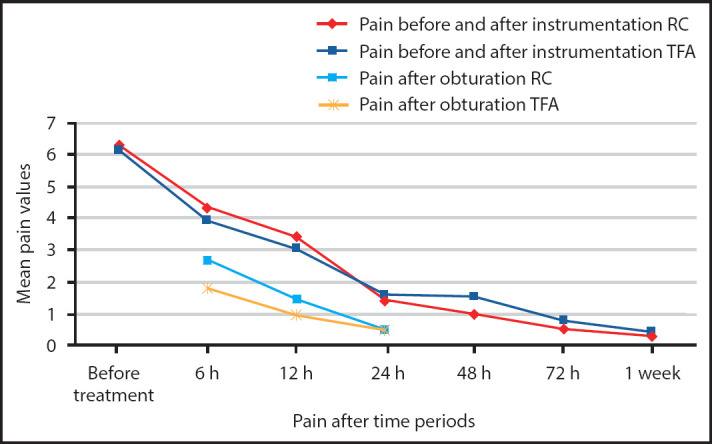
Line chart for mean pain values on NRS before and after instrumentation and after obturation of RC and TFA RC: Reciproc, TFA: TF Adaptive

The demographic data showed no significant difference in both groups and are summarised in Table 1. Regarding post-instrumentation pain, there was no statistically significant difference (P>0.05) between RC and TFA groups in mean values and quality of pain severity at each time interval using the Mann-Whitney U test (Tables 2, 3, and Fig. 1). In each group, pain decreased with a statistically significant difference from preoperative condition to post-instrumentation after 6, 12, 24, 48, 72 hrs, and 1 week (P<0.001). There was a statistically significant difference between pain after 6 hrs or after 12 hrs compared to other time intervals (P<0.001). There was no statistically significant difference between pain values after 24 and 48 hrs, and also, no significant difference was found between 72 hrs and 1 week (P>0.05).

**TABLE 1.** Descriptive and statistical analysis of demographic data of both groups

Instrumentation system	RC	TFA	P
Age			
Mean (SD)	31.28 (12.56)	30.21 (10.93)	**0.731**
Gender, n (%)			
Female	23 (79.3)	20 (69)	**0.372**
Male	6 (20.7)	9 (31)	
Periapical lesion, n (%)			
Yes	25 (86.2)	22 (75.9)	**0.319**
No	4 (13.8)	7 (24.1)	
Apical periodontitis, n (%)			
Symptomatic	27 (93)	26 (89.6)	**0.899**
Asymptomatic	2 (6.8)	3 (10.3)	

Significant (P<0.05). RC: Reciproc, TFA: TF adaptive, SD: Standard deviation, n: Number of patients

**TABLE 2.** Descriptive and statistical analysis of pain before and after instrumentation and after obturation of RC and TFA

Variables	RC		TFA		P
	Mean (SD)	Range	Mean (SD)	Range	
Preoperative pain	6.28 (3.34)^a^	0-10	6.14 (3.82)^a^	0-10	0.969
Pain after 6 hrs.	4.31 (3.86)^b^	0-10	3.90 (3.53)^b^	0-10	0.555
Pain after 12 hrs.	3.41 (3.39)^c^	0-10	3.03 (3.23)^c^	0-10	0.663
Pain after 24 hrs.	1.41 (2.13)^d^	0-7	1.59 (2.29)^d^	0-8	0.785
Pain after 48 hrs.	1.00 (1.89)^d^	0-6	1.55 (2.15)^d^	0-7	0.192
Pain after 72 hrs.	0.52 (1.45)^e^	0-7	0.79 (1.68)^e^	0-6	0.489
Pain after 1 week	0.31 (1.17)^e^	0-6	0.41 (0.82)^e^	0-3	0.124
P-value	<0.001*		<0.001*		
Pain after obturation					
Pain after 6 hrs.	2.69 (3.62)^a^	0-10	1.79 (2.55)^a^	0-7	0.428
Pain after 12 hrs.	1.48 (2.34)^b^	0-7	0.97 (1.61)^ab^	0-5	0.395
Pain after 24 hrs.	0.48 (1.60)^b^	0-7	0.48 (1.21)^b^	0-5	0.720
P-value	<0.001*		<0.001*		

*: Significant (P<0.05), same superscript letters indicates no significant difference, RC: Reciproc, TFA: TF Adaptive

**TABLE 3.** Number and percentage of patients with no, mild, moderate, or severe pain based on NRS before and after instrumentation and after obturation; preoperatively, after 6, 12, 24, 48, 72 hrs, and 1 week

	Pain before and after instrumentation				
Pain quality*	RC		TFA		P
	n	(%)	n	(%)	
Preoperative pain					
No	2	(6.8)	3	(10.3)	0.952
Mild	4	(13.7)	5	(17)	
Moderate	8	(27.5)	5	(17)	
Severe	15	(51.7)	16	(55)	
6 hrs					
No	6	(20.6)	9	(31)	0.859
Mild	10	(34.5)	4	(13.7)	
Moderate	3	(10.3)	7	(24)	
Severe	10	(34.5)	9	(31)	
12 hrs					
No	8	(27.5)	10	(34.5)	0.518
Mild	10	(34.5)	8	(27.5)	
Moderate	3	(10.3)	7	(24)	
Severe	8	(27.5)	4	(13.7)	
24 hrs					
No	18	(62)	17	(58.6)	0.696
Mild	6	(20.6)	5	(17)	
Moderate	4	(13.7)	6	(20.6)	
Severe	1	(3.4)	1	(3.4)	
48 hrs					
No	20	(69)	15	(51.7)	0.220
Mild	5	(17)	9	(31)	
Moderate	4	(13.7)	4	(13.7)	
Severe	0	(0)	1	(3.4)	
72 hrs					
No	24	(82.7)	22	(75.8)	0.488
Mild	4	(13.7)	4	(13.7)	
Moderate	0	(0)	3	(10.3)	
Severe	1	(3.4)	0	(0)	
1 week					
No	26	(89.6)	21	(72.4)	0.117
Mild	2	(6.8)	8	(27.5)	
Moderate	1	(3.4)	0	(0)	
Severe	0	(0)	0	(0)	
Pain after obturation					
6 hours					
No	15	(51.7)	17	(58.6)	0.291
Mild	3	(10.3)	5	(17)	
Moderate	5	(17)	6	(20.6)	
Severe	6	(20.6)	1	(3.4)	
12 hours					
No	17	(58.6)	17	(58.6)	0.367
Mild	7	(24)	7	(24)	
Moderate	4	(13.7)	4	(13.7)	
Severe	1	(3.4)	1	(3.4)	
24 hours					
No	25	(86)	24	(82.7)	0.738
Mild	3	(10.3)	4	(13.7)	
Moderate	0	(0)	1	(3.4)	
Severe	1	(3.4)	0	(0)	

*: Significant (P<0.05). RC: Reciproc, TFA: TF Adaptive, n: Number of patients

There was no statistically significant difference between the two groups at 6, 12, and 24 hrs regarding pain after obturation. In each group, pain decreased progressively from 6 to 24 hrs. There was a significant difference between pain at 6 hours and pain at 12 and 24 hrs (P<0.001, Tables 2, 3, and Fig. 1).

Comparing pain scores after instrumentation versus after obturation, mean pain values at 6, 12, and 24 hrs after instrumentation was higher than pain values at 6, 12, and 24 hrs after obturation with a statistically significant difference in both groups (P<0.001).

The symptomatic and asymptomatic patients were equally distributed among the two groups (Table 1). However, there was a statistically significant difference between the number of symptomatic and asymptomatic cases within each group (P<0.001).

Pearson correlation value (r) of preoperative pain revealed a significant positive relationship with pain at 6 (P=0.003), 12 (P<0.001), and 24 hours after instrumentation (P=0.001). Durations of total active instrumentation of TFA group had higher mean value than RC group, 9.21 min, and 8 min; respectively with no statistically significant difference between two groups (P=0.111). In addition, the mean analgesic intake was higher in the TFA group (2.48 tablets) than in the RC group (2.41 tablets), with no statistically significant difference between the two groups (P=0.339).

## DISCUSSION

It is reported that different file designs, kinematics, and systems may impact postoperative pain (15). Caviedes-Bucheli et al., (16) in a systematic review, concluded that the amount of neuropeptide expression was higher in teeth where the root canals were prepared with a reciprocating motion by WaveOne than other rotary file systems. Thus, in the present study, pain was assessed after instrumentation using RC files with reciprocating motion versus TFA system with adaptive motion on necrotic mandibular molars in a randomised clinical trial design. Randomisation and allocation concealment was done to eliminate the effect of selection bias.

The two-visit protocol of the current study eliminated the confounding effects of type and extent of obturating materials. The two-visit protocol also allowed the chance for emergency treatment in cases of flare-ups or swelling. Of note, no intracanal dressing was used between the appointments as it was reported that as long as proper cleaning and shaping were performed, a non-significant difference existed between the presence or absence of intra-canal medication (17). Additionally, eliminating intracanal medication avoided the possible confounding effect on postoperative pain (18).

Teeth with necrotic pulps were selected, as it is reported that the most likely predisposing clinical condition for the occurrence of postoperative pain is necrotic pulp with a periapical lesion (1). Symptomatic patients were included in the study as they present more challenges because preoperative pain is established to be a predisposing factor of postoperative pain (19). Asymptomatic necrotic patients were also included due to their increased likelihood of flare-up that might occur after instrumentation (20).

According to the present study's results, the null hypothesis was accepted as there was no statistically significant difference between the two instrumentation techniques regarding post-instrumentation pain. Also, there was no clinical significance between groups.

Recently, systematic review and meta-analysis (21) found no difference in postoperative pain after using rotary or reciprocating instruments. However, all included studies assessed post-obturation pain as they were conducted in a single session, except for two articles. Thus, post-instrumentation pain values at each time interval for the RC group were compared with the clinically available two studies; Oliveira et al. (8) on RC and Nekoofar et al. (5) on WaveOne. In the present study, after 24 hrs, the mean post-instrumentation value of RC was almost similar to that reported by Oliveira et al. (8) using RC for instrumenting molars diagnosed with irreversible pulpitis. However, Nekoofar et al. (5) reported the mean post-instrumentation pain for teeth with irreversible pulpitis after five intervals; 6, 12, 24, 48 hrs, and 72 hrs was slightly lower than the values of the present study. This difference between the two studies may be explained by their diagnosis of irreversible pulpitis versus necrotic teeth in the current study. Furthermore, they used chlorhexidine as an irrigant versus NaOCl in the present study. In addition, their reciprocating instrument was WaveOne.

For post-instrumentation pain within each group, both groups' greatest mean pain value occurred in the first 24 hrs, especially at 6, 12 hrs after instrumentation. This may be attributed to the amounts of neuropeptides expression as substance P and calcitonin gene-related peptide, leading to the sensitisation or activation of neurons causing pain (16). The amounts of extruded debris and neuropeptides released from C-fibers found in the periodontal ligament differ with the use of different instrumentation techniques (16). There was a significant reduction in pain ratings at the subsequent observation time points of 48, 72 hrs, and 7 days similar to previous reports (5, 6). Worthy to note, observing the mean of pain at 6, 12 hrs in the current study, it ranged approximately between scores 3 to 4 in both groups, thus giving an impression of swinging between mild-moderate tolerable pain.

In the present study, after 24 hrs, post-obturation pain in the RC group reported a high percentage, 86% of patients having no pain almost similar to Relvas et al. (22), who reported 84% of patients with no pain with, however in cases of asymptomatic pulp necrosis. On the other hand, in a prospective randomised multicenter study by Neelakantan et al. (7), 71.4% of patients had mild pain. Additionally, Zand et al. (12) reported a significantly lower percentage of mild or pain-free patients (33%). Moderate postoperative pain occurred in 64% of their patients.

The present study's results regarding TFA can be compared to a clinical study by Gambarini et al. (10) that reported post-obturation pain 24 hrs after a single-visit RCT using TFA to be 46.6%. In the current study, after 24 hrs, the percentage of patients with pain after obturation was 17.3% in the TFA group, almost half the study mentioned above (10). This may be attributed to differences in the type of teeth as they included maxillary and mandibular molars and premolars versus mandibular molars in the present study. Also, they used warm vertical compaction, and the present study used the lateral compaction technique. Finally, they irrigated canals with open-ended needles versus side-vented needles.

The present study showed that the post-instrumentation pain was statistically higher than post-obturation pain in both groups. A similar observation was revealed by Nekoofar et al. (5). This result confirmed that instrumentation of the canals had a greater effect on pain than post obturation probability due to extruding dentine debris and microorganisms beyond the apex (23).

The duration of total active instrumentation with RC or TFA showed no statistically significant difference between the two groups. The mean duration of instrumentation using the single file RC group was 8 min (480 sec), and in the three-file TFA system was 9.21 min (552 sec). Although there was no clinical significance between groups, both times were within the clinical comfort for the patient and clinician. Notably, the single file RC approached the time for the three-file TFA system. This may be due to the difficulty of RC files to reach full working length than TFA files. RC needed frequent irrigation between each stroke and confirmation of patency of the canals frequently, which needed more time. On the contrary, though TFA consists of 3 files with less taper, it could reach full working length easier than RC. Yilmaz and Ozyurek (24) showed similar findings, who found no statistically significant difference between TFA and RC in instrumentation duration.

As for the number of analgesics, there was no statistically significant difference between the two groups in the mean number of analgesic tablets taken along all-time intervals. Similarly, Arslan et al. (25) found no statistically significant difference in the number of analgesic tablets taken after using RC in reciprocation or rotation. Their mean number of analgesic tablets taken in the RC group was two, similar to the present study. It should be noted that all patients who experienced moderate or severe post-obturation pain also had moderate or severe post-instrumentation pain, and most of them have taken analgesics.

The external validity of the present study was affected by the limited availability of TFA in the markets after widespread heat-treated NiTi alloys. In addition, in the present study, the small number of asymptomatic patients and strict eligibility criteria for selecting only necrotic cases added limitations to the study. Therefore, a cohort study with an appropriate sample size might be recommended to test the effect of symptomatic versus asymptomatic cases when using different file systems on post-instrumentation and post-obturation pain.

## CONCLUSION

Within the limitation of the present study, it was concluded that TFA and RC had a similar impact on post-instrumentation and post-obturation pain. However, post-instrumentation pain was higher than post-obturation pain in both groups. Therefore, further studies are needed to determine the effect of using adaptive motion with different file systems on post-instrumentation pain in different pulp and periapical diseases.
